# S-Propargyl-cysteine Exerts a Novel Protective Effect on Methionine and Choline Deficient Diet-Induced Fatty Liver via Akt/Nrf2/HO-1 Pathway

**DOI:** 10.1155/2016/4690857

**Published:** 2016-05-22

**Authors:** Wenwen Li, Fenfen Ma, Laiyin Zhang, Yong Huang, Xinghui Li, Aijie Zhang, Cuilan Hou, Yichun Zhu, YiZhun Zhu

**Affiliations:** ^1^Shanghai Key Laboratory of Bioactive Small Molecules and Research Center on Aging and Medicine, Department of Physiology and Pathophysiology, Shanghai Medical College, Fudan University, Shanghai 200032, China; ^2^Department of Pharmacology, School of Pharmacy, Fudan University, Shanghai 201203, China; ^3^Department of Pharmacy, Linyi People's Hospital, Linyi 276000, China; ^4^Department of Pharmacology, Yong Loo Lin School of Medicine, National University of Singapore, Singapore 119077

## Abstract

This study investigated the antioxidative effect of S-propargyl-cysteine (SPRC) on nonalcoholic fatty liver (NAFLD) by treating mice fed a methionine and choline deficient (MCD) diet with SPRC for four weeks. We found that SPRC significantly reduced hepatic reactive oxygen species (ROS) and methane dicarboxylic aldehyde (MDA) levels. Moreover, SPRC also increased the superoxide dismutase (SOD) activity. By Western blot, we found that this protective effect of SPRC was importantly attributed to the regulated hepatic antioxidant-related proteins, including protein kinase B (Akt), heme oxygenase-1 (HO-1), nuclear factor erythroid 2-related factor 2 (Nrf2), and cystathionine *γ*-lyase (CSE, an enzyme that synthesizes hydrogen sulfide). Next, we examined the detailed molecular mechanism of the SPRC protective effect using oleic acid- (OA-) induced HepG2 cells. The results showed that SPRC significantly decreased intracellular ROS and MDA levels in OA-induced HepG2 cells by upregulating the phosphorylation of Akt, the expression of HO-1 and CSE, and the translocation of Nrf2. SPRC-induced HO-1 expression and Nrf2 translocation were abolished by the phosphoinositide 3-kinase (PI3K) inhibitor LY294002. Moreover, the antioxidative effect of SPRC was abolished by CSE inhibitor DL-propargylglycine (PAG) and HO-1 siRNA. Therefore, these results proved that SPRC produced an antioxidative effect on NAFLD through the PI3K/Akt/Nrf2/HO-1 signaling pathway.

## 1. Introduction

Nonalcoholic fatty liver disease (NAFLD) that is currently one of the most common chronic liver diseases is considered to be closely associated with central obesity, dyslipidemia, hypertension, hyperglycemia, and other metabolic disorders [1]. The theory of the “two hits” hypothesis proposed by Day and James indicated that oxidative stress (OS) and dysregulation of redox-sensitive signaling pathways were vital to the pathobiology of fatty liver diseases [2, 3]. Reactive oxygen species (ROS) is mainly responsible for the OS control, which is extremely important to the cell homeostasis [4]. Therefore, pharmacological approaches for intervening in OS might be therapeutic intervention strategies for NAFLD.

HO-1 is the most important rate-limiting enzyme in heme catabolism and can strongly protect the liver from oxidative damage and cell death [5, 6]. Previous studies indicated that the expression of HO-1 in the liver was generally regulated by the transcription factor nuclear factor erythroid 2-related factor 2 (Nrf2) [7]. Moreover, recent studies indicated a close link between phosphoinositol 3-kinase (PI3K/Akt) and Nrf2 activation [8, 9]. Accordingly, we hypothesize that the phosphorylation of Akt, translocation of Nrf2, and subsequent modulation of HO-1 expression were considered as the important molecular targets for therapeutic intervention for NAFLD [10].

S-Propargyl-cysteine (SPRC, ZYZ-802) is a structural analog of S-allylcysteine (SAC) that is the most abundant constituent of aged garlic extract. Due to the cysteine structure, SPRC is recognized as the substrate for endogenous H_2_S synthesis via CSE catalysis [11–16]. In addition, previous studies have proven that SPRC is a novel synthetic molecule that exerts antioxidative and anti-inflammatory effects via modulating the endogenous H_2_S level and activating Nrf2 translocation in H9C2 cells [17, 18]. However, the effects of SPRC on NAFLD and the underlying mechanism remain unclear. Therefore, this study investigated the role and possible mechanisms of SPRC on MCD diet-induced NAFLD.

## 2. Materials and Methods

### 2.1. Materials

S-Propargyl-cysteine (SPRC, also named as ZYZ-802) was synthesized from the reaction of L-cysteine with propargyl bromide and purified by recrystallization from an ethanol-water mixture (96.1%) as described previously [12]. The final product was verified by ^1^H nuclear magnetic resonance spectroscopy. The product purity was over 99%, which is determined by high performance liquid chromatography. Oil red dye, oleic acid (OA), DL-propargylglycine (PAG), dihydroethidium (DHE), and LY294002 were purchased from Sigma-Aldrich (St. Louis, MO, USA). Fatty-acid-free bovine serum albumin (FAF-BSA) was obtained from Merck (Germany). Detection kits for methane dicarboxylic aldehyde (MDA), triglyceride (TG), and cholesterol (TC) levels were purchased from Beyotime Biotechnology Corp. and Nanjing Jiancheng Bioengineering Institute (China). The Takara quantitative RT-PCR kit and SYBR Green Premix were products of Takara Biomedicals Inc. (Shiga, Japan). The primary and secondary antibodies used for Western blot [phosphorylation-Akt, Akt, Nrf2, and glyceraldehyde-3-phosphate dehydrogenase (GAPDH)] were purchased from Cell Signaling Technology (Beverly, MA, USA). HO-1 and CSE primary antibody were bought from ProteinTech Group, Inc. (Chicago, IL, USA). Nuclear and cytoplasmic proteins of HepG2 cells were extracted using the NE-PER® Nuclear and Cytoplasmic Extraction Reagents (Pierce Inc.) according to manufacturer's instructions.

### 2.2. Animals Model

Male C57 BL/6 mice (8 weeks old) were purchased from Sippr-BK Experimental Animal Center (Shanghai, China) and raised at standard condition of animal housing (12-hour light/dark cycle; temperature 25°C; humidity 55–60%). Mouse model of MCD diet-induced NAFLD was duplicated according to the previous studies [19–21]. After one-week adaptive feeding, 60 mice were randomly divided into normal control group, model control group, and different dosages of SPRC groups. The mice in the normal control group got free access to the methionine/choline-supplement (MCS) diet (Trophic Animal Feed High-Tech Co., China), and mice in the MCD control group, MCD+SPRC group, and MCD+SPRC+PAG group were all fed with a MCD diet (Trophic Animal Feed High-Tech Co., China) for four weeks. Mice in the SPRC 20, 40, and 80 mg/kg or PAG 50 mg/kg+SPRC 40 mg/kg/d intervention groups were intraperitoneally injected with SPRC or PAG daily. Mice in the MCS control group and MCD control group received the same volume of saline solution. Note that the SPRC+PAG group was used to prove whether the effect of SPRC depends on CSE activity. After the treatment of four weeks, all mice were sacrificed with the livers and blood samples isolated for further study. All animals were handled according to the* Guide for the Care and Use of Laboratory Animals* published by the US National Institutes of Health (NIH). Experimental procedures were managed according to the local ethical committee of Fudan University.

### 2.3. Biochemical Analyses

At the end of the experimental period, mice were fasted for 12 h and then euthanized with pentobarbital sodium. Blood samples were collected, and plasma was then prepared by centrifuging the blood samples at 3000 rpm at 4°C for 15 min. The plasma levels of alanine transaminase (ALT) and aspartate transaminase (AST) were determined by automatic biochemical analyzer (Cobas 6000, Roche). SOD activities and TG, TC, and MDA levels in mice livers were determined by commercially available kits after homogenization (Beyotime Biotechnology Corp.; Nanjing Jiancheng Bioengineering Institute, China). Protein concentrations of tissues were assayed by the BCA method. SOD activity/TG/TC/MDA levels were normalized to protein contents and presented as fold of control for* in vivo* studies.

### 2.4. Morphological and Histological Analyses

Liver was surgically removed to determine the weight ratio of liver to body (LW/BW × 100%). For histological analysis, the liver was excised, fixed in 10% formalin for 48 h before dehydration using a graded ethanol series, embedded in paraffin, sectioned at 4 *μ*m thickness, and stained with hematoxylin and eosin (H&E). ROS level in mice livers was detected by dihydroethidium (DHE, Sigma-Aldrich) fluorescence probe. Liver tissue was embedded by optimal cutting temperature compound (O.C.T. compound, SAKURA, USA) and cut into sections (15 *μ*m). Sections were incubated with DHE (10 *μ*mol/L) at 37°C for 30 min in the dark. Excess DHE was then removed by PBS-washing. Fluorescence was observed and images were captured using fluorescence microscope (ZEISS AX10, Germany).

### 2.5. Western Blot Analyses

Liver tissue or cells were lysed in lysis buffer for 10 min at 4°C. Protein concentration was determined with a BCA protein assay kit (Shen Neng Bo Cai Corp., China). Equal amounts (30 *μ*g) of protein were separated on 10% sodium dodecyl sulfate-polyacrylamide gels and transferred to nitrocellulose membrane (Millipore). After being blocked with 5% nonfat dry milk, membranes were incubated overnight at 4°C with Akt, phosphorylated Akt (Ser473), Nrf2, GAPDH antibodies (Cell Signaling Technology, Inc., Beverly, MA, USA), CSE, and HO-1 antibodies (ProteinTech, Inc., USA). Horse radish peroxidase- (HRP-) conjugated goat anti-rabbit or goat anti-mouse IgG (ICL Lab, Newberg, OR, USA) was used as a secondary antibody. Specific bands were detected with SuperSignal West Pico Chemiluminescent Substrate (Thermo Scientific Pierce).

### 2.6. Plasma H_2_S Concentration Assay

The concentration in plasma was determined as previously described [22]. Monobromobimane (MBB) reacts with hydrogen sulfide under basic conditions to produce sulfide-dibimane. Sulfide-dibimane is more hydrophobic than most physiological thiols. This characteristic allows sulfide-dibimane to be separated with a gradient elution and analyzed by fluorescence detection. Blood samples were obtained from abdominal aorta when the animals were sacrificed. Prior to the detection, the MBB stock and calibration standard were prepared. Samples pretreated with 20 *μ*L anticoagulation [15% ethylene diamine tetraacetic acid (EDTA)] were centrifuged at 3000 rpm for 15 min at 4°C to obtain plasma. For derivatization reaction of hydrogen sulfide with MBB, add 30 *μ*L plasma, 80 *μ*L MBB, and 10 *μ*L 0.1% ammonia to each sample in 1.5 mL tubes, shaking for 1 h. 10 *μ*L 20% formic acid was added to terminate the reaction. Samples were centrifuged again at 15,000 g for 10 min and the supernatants (60 *μ*L) that contain the fluorescent product sulfide-dibimane (SDB) were detected by Liquid Chromatography-Mass Spectrometry (LC-MS).

### 2.7. Cell Culture and Treatment

HepG2 cells were cultured in Modified Eagle's Medium (MEM, Life Technologies) supplemented with 10% (v/v) fetal bovine serum (FBS, Thermo Fisher Hyclone), penicillin/streptomycin (100 units) (Life Technologies), and nonessential amino acid (NEAA, 100×, Life Technologies) maintained in an incubator (37°C with 5% CO_2_). Indicated concentration of oleic acid was prepared with 1% FAF-BSA solvent. Cells were then cultured in medium without oleic acid (OA), which served as a nontreated control (NC) group, or with OA (1.5 mmol/L, control group) or SPRC+OA (SPRC) group. In indicated experiments, cells were also needed to preadd PAG or LY 294002 in the medium of the SPRC-treated group, and the control groups were treated with the vehicle.

### 2.8. Cell Viability Assays

Cell viability of HepG2 cells cultured in 96-well plates was measured using the Cell Counting Kit-8 (CCK-8) (Dojindo Molecular Technologies) according to the manufacturer's instructions. The absorbance of CCK-8 was obtained at 450 nm with a microplate reader. The values were normalized to the nontreated (NC) group.

### 2.9. Assay for Intracellular ROS, TG, MDA, and SOD Activity

ROS level in HepG2 was determined by dihydroethidium (DHE, Sigma-Aldrich) fluorescence using confocal microscopy. After treatments for 24 h, cells were washed with PBS and incubated with DHE (10 *μ*mol/L) at 37°C for 30 min in the dark. Then, DHE was removed by PBS-washing [23]. Fluorescence was observed by a microplate reader (excitation, 488 nm; emission, 610 nm) (Tecan, Austria) or under a laser confocal microscope (Zeiss LSM710, Germany). The values were normalized to the nontreated (NC) group. After treatment, SOD activity and TG and MDA levels in cells were determined by commercially available kits after homogenization (Beyotime Biotechnology Corp.; Nanjing Jiancheng Bioengineering Institute, China). Protein concentrations of cells and tissues were assayed by the bicinchoninic acid (BCA) method. SOD activity and TG and MDA levels were normalized to protein contents and presented as fold of control for* in vitro* studies.

### 2.10. Oil Red Staining

Oil red O staining was performed as mentioned before [24]. In general, treated HepG2 cells were washed twice with PBS and fixed with 4% paraformaldehyde for 30 min at room temperature. Cells were then washed by PBS, followed by staining with oil red O for 1 h at room temperature. Cells were washed and extracted by using 100% isopropanol. Pictures were taken with Leica microscope in combination with a digital camera at ×200 magnifications. OD was measured at 510 nm wave length using a microplate reader (Tecan, Austria).

### 2.11. Quantitative Real-Time PCR

Total RNA was extracted by the TRIZOL reagent (Gibco, USA). First strand cDNA was synthesized with PrimeScript RT Master Mix (Takara, Japan) according to the manufacturer's instructions. The quantitative real-time PCR was performed on a step-one plus real-time PCR detection system (Applied Biosystems, USA) by using SYBR Premix (Takara, Japan) according to the manufacturer's instructions. To investigate the effects of SPRC on ROS-related genes, the mRNA expression of Akt, HO-1, Nrf2, and CSE was examined. Primer sequences were as follows: Akt (5′-GCACCTTCCATGTGGAGACT-3′ and 5′-CCCAGCAGCTTCAGGTACTC-3′); HO-1 (5′-ACTGCGTTCCTGCTCAACAT-3′ and 5′-GGGCAGAATCTTGCACTTTGT-3′); Nrf2 (5′-CCAACTACTCCCAGGTTGCC-3′ and 5′-AAGTGACTGAAACGTAGCCGA-3′); CSE (5′-AGCATGCAGGAAAAAGACGC-3′ and 5′-ATATTCAAAACCCGAGTGCTGG-3′); GAPDH (5′-TGGTCACCAGGGCTGCTTTTA-3′ and 5′-TCCTGGAAGATGGTGATGGGATTT-3′). Real-time PCR conditions were one cycle of 95°C for 15 min, followed by 40 cycles of 95°C cDNA denaturation for 10 s, 60°C primer annealing for 30 s, and 72°C extension for 30 s. The expression levels of each gene were normalized against GAPDH using the comparative 2^−ΔΔCT^ method and the results were from three independent experiments according to the manufacturer's protocols [25].

### 2.12. siRNA Transfection

HepG2 cells were transfected with human HO-1 siRNA (sc-35554, Santa Cruz Biotechnology) using Lipofectamine 3000 (Invitrogen) and Opti-MEM medium (Gibco). Briefly, HepG2 cells were trypsinized and plated on 6-well plates at 30% to 50% confluence 24 h before transfection. HO-1 siRNA (50 nmol/L), Lipofectamine 3000, and Opti-MEM were mixed and incubated at room temperature for 20 min. siRNA-Lipofectamine 3000 complexes were added and the medium was replaced with fresh MEM medium after transfection for 6 h. Experiments were performed 24 h after transfection. Knockdown of HO-1 was assessed by Western blot. Intracellular ROS level was observed using DHE probe under a laser confocal microscope (Zeiss LSM710, Germany).

### 2.13. Data Analyses

All the data were presented as means ± SEM. Differences were compared by one-way ANOVA analysis by using SPSS software version 19.0 (SPSS Inc., Chicago, IL, USA). And *P* value < 0.05 was taken as statistically significant.

## 3. Results

### 3.1. The Effect of SPRC on Liver Injury in MCD Diet-Induced NAFLD Mice

**Figure 1:**
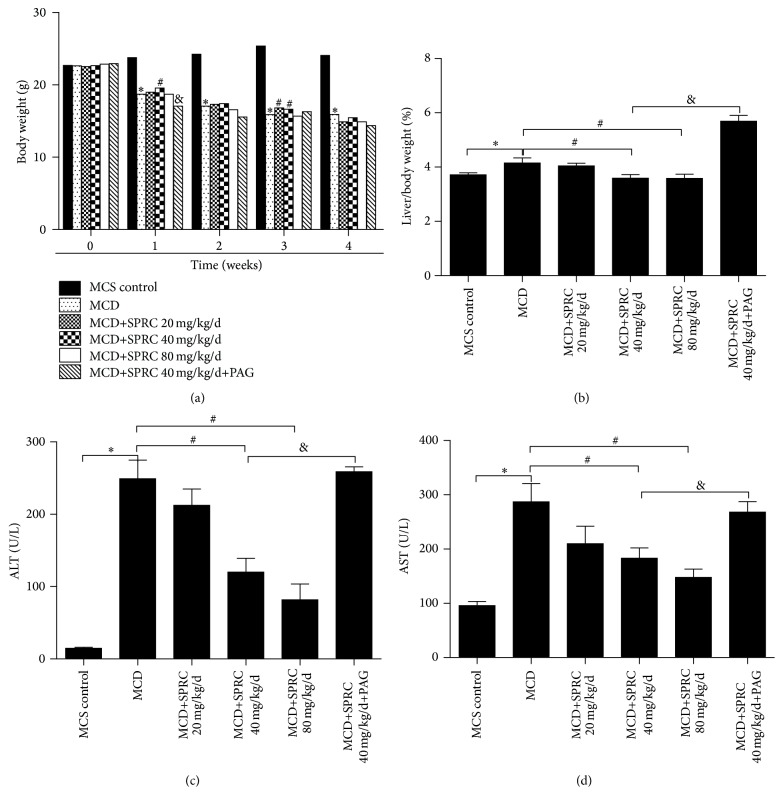
The effect of SPRC on liver injury of NAFLD mice fed MCD diet. Mice fed a MCD diet were treated with SPRC (20, 40, and 80 mg/kg/d) and SPRC+PAG for four weeks. (a) The effect of SPRC and PAG on body weight. (b) The effect of SPRC and PAG on liver/body ratio. (c) The effect of SPRC on plasma ALT. (d) The effect of SPRC on plasma AST. Data shown are means ± SEM; ^*∗*^ *P* < 0.05 compared with MCS diet control group, *n* = 8; ^#^ *P* < 0.05 compared with MCD diet control group, *n* = 8; ^&^ *P* < 0.05 compared with MCD+SPRC 40 mg/kg/d+PAG group, *n* = 8.

To investigate the* in vivo* effect of SPRC on NAFLD, mice were fed a MCD diet for four weeks. As shown in Figure 1(a), four weeks of MCD diet feeding dramatically decreased the body weight resulting from malnutrition. However, groups treated with SPRC (40 mg/kg/d) significantly regained the weight at the 1st week, and the SPRC (20, 40 mg/kg/d) groups also alleviated the weight loss at the 3rd week compared to the MCD control group. Nevertheless, the data showed that PAG reversed the effect on the weight loss only at the 1st week compared to SPRC 40 mg/kg/d group. When the liver suffers from injury, liver organ index (liver/body weight) becomes higher. In our study, MCD diet caused severe damage to the liver and increased liver/body weight ratio, while SPRC intervention caused a distinct decrease in the liver/body weight ratio. In particular, even if we treated MCD diet-induced mice with SPRC (40 mg/kg/d) in the PAG+SPRC group, PAG treatment still reversed this effect and the protective effect of SPRC was abolished compared to the SPRC (40 mg/kg/d) group (Figure 1(b)). Aspartate transaminase (ALT) and alanine transaminase (AST) are generally determined to evaluate hepatocellular injury. As displayed in Figures 1(c) and 1(d), the graphs indicated that MCD diet led to a dramatic increase in plasma ALT and AST levels, while SPRC treatment remarkably suppressed the ALT and AST elevation. All the effects of SPRC were reversed in the SPRC+PAG group, as shown in Figure 1.

### 3.2. The Protective Effect of SPRC on Hepatic MDA Level, SOD Activity, Lipid Accumulation, and ROS Level in MCD Diet-Induced NAFLD Mice

**Figure 2:**
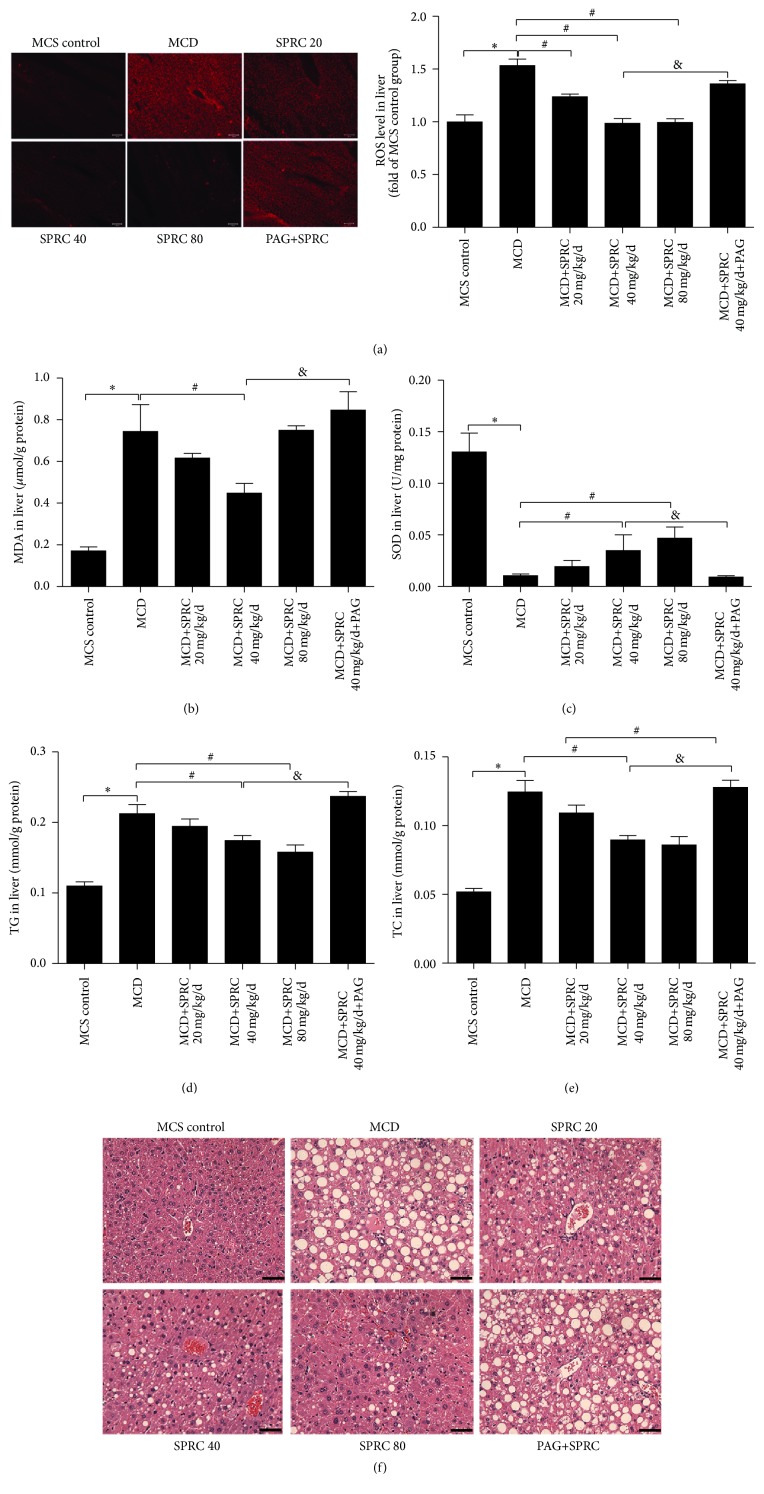
The antioxidant effect of SPRC on hepatic ROS level, MDA level, SOD activity, and lipid content in the livers of NAFLD mice fed MCD diet. Mice fed a MCD diet were treated with SPRC (20, 40, and 80 mg/kg/d) and SPRC+PAG for four weeks. (a) The effect of SPRC on hepatic ROS level. Photomicrographs were taken at ×100 magnification. (b) The effect of SPRC on hepatic MDA level. (c) The effect of SPRC on hepatic SOD activity. (d) The effect of SPRC on hepatic TG level. (e) The effect of SPRC on hepatic TC level. (f) Representative images for the liver stained by H&E (original magnification, ×200; scale bar = 250 *μ*m). Data shown are means ± SEM; ^*∗*^ *P* < 0.05 compared with MCS diet control group, *n* = 8; ^#^ *P* < 0.05 compared with MCD diet control group, *n* = 8; ^&^ *P* < 0.05 compared with MCD+SPRC 40 mg/kg/d+PAG group, *n* = 8.

To verify the antioxidative and lipid regulating effects of SPRC, we detected the hepatic MDA level, SOD activity, and TG and TC levels using chemical kits. ROS level was observed by fluorescence microscope. As shown in Figure 2(a), four weeks of MCD diet dramatically increased ROS level, while groups treated with SPRC (20, 40, and 80 mg/kg/d) significantly reduced ROS level. In particular, even if we treated MCD diet-induced mice with SPRC (40 mg/kg/d) in the PAG+SPRC group, PAG treatment still reversed the effect of SPRC on ROS reducing compared to the SPRC (40 mg/kg/d) group. Four-week MCD diet also caused sharp increase of MDA and decrease of SOD activity compared to the MCS control group. However, SPRC (40 mg/kg/d) treatment remarkably reduced the hepatic MDA level (Figure 2(b)). SPRC (20, 40 mg/kg/d) treatment also enhanced hepatic SOD activity (Figure 2(c)). In addition, as shown in Figures 2(d) and 2(e), MCD diet feeding induced serious liver injury, resulting in an excess of lipid accumulation (TG, TC), while SPRC (40, 80 mg/kg/d) downregulated the TG level, and SPRC (40 mg/kg/d) treatment also reduced TC level. In Figure 2(f), H&E staining revealed severe steatosis with a mass of vacuoles in the livers of mice fed with MCD diet, which was alleviated when treated with SPRC. Finally, all the effects of SPRC were abolished in the SPRC+PAG group, as shown in Figure 2.

### 3.3. Effect of SPRC on the Expressions of Antioxidant-Related Proteins in Mice Livers

**Figure 3:**
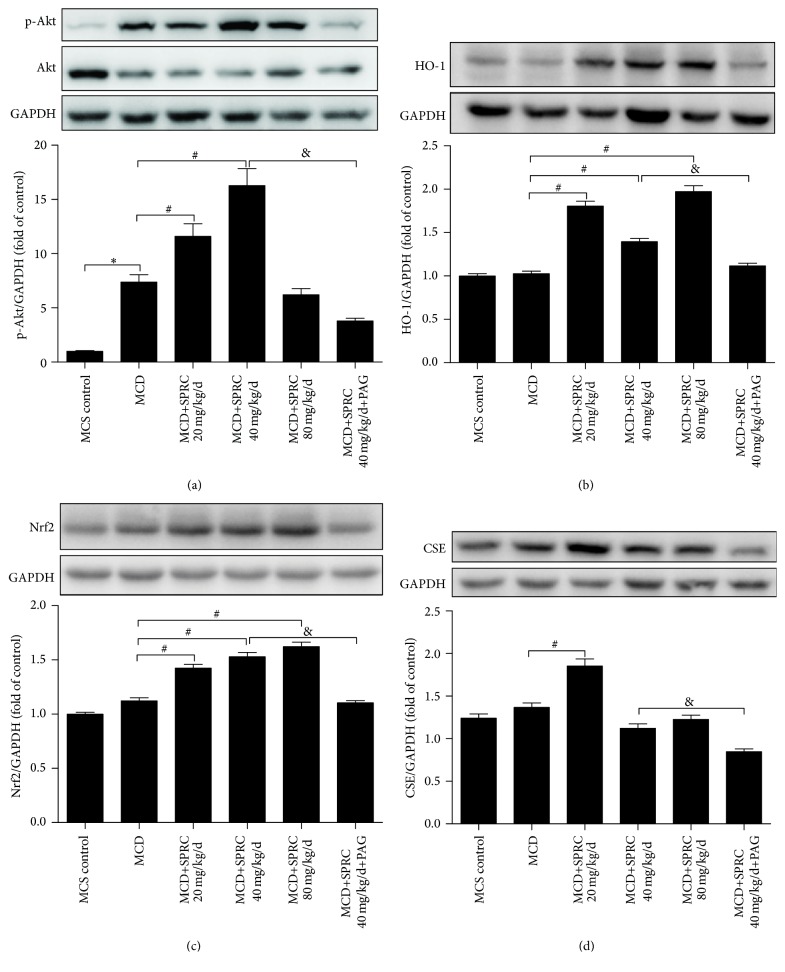
The effect of SPRC on the expression of antioxidant-related proteins in the mouse liver tissue. Mice fed a MCD diet were treated with SPRC (20, 40, and 80 mg/kg/d) and SPRC+PAG for four weeks. (a) Western blot analysis for the phosphorylation of Akt. (b) Western blot analysis for the expression of HO-1. (c) Western blot analysis for the expression of Nrf2. (d) Western blot analysis for the expression of CSE. GAPDH was used as loading control; data shown are means ± SEM; ^*∗*^ *P* < 0.05 compared with MCS diet control group, *n* = 8; ^#^ *P* < 0.05 compared with MCD diet control group, *n* = 8; ^&^ *P* < 0.05 compared with MCD+SPRC 40 mg/kg/d+PAG group, *n* = 8.

The expression and phosphorylation levels of several signaling elements were examined by the Western blot analysis in liver tissue at the end of the four-week SPRC treatment. A certain degree of increase of Akt phosphorylation was detected in the MCD control group, compared to the MCS control group. However, Akt phosphorylation was significantly increased with SPRC at dosages of 20 and 40 mg/kg/d, but not at the dosage of 80 mg/kg/d, compared with the MCD control group (Figure 3(a)). And in the PAG group, this effect was abolished. As shown in Figures 3(b) and 3(c), the expressions of HO-1 and Nrf2 were remarkably upregulated by SPRC treatment (20, 40, and 80 mg/kg/d). The expression of CSE was also significantly increased in the SPRC (20 mg/kg/d) group (Figure 3(d)). In addition, all the effects of SPRC displayed in Figure 3 were reversed in the SPRC+PAG group compared to the MCD+SPRC 40 mg/kg/d group.

### 3.4. The Effect of SPRC on Endogenous H_2_S Concentration

**Figure 4:**
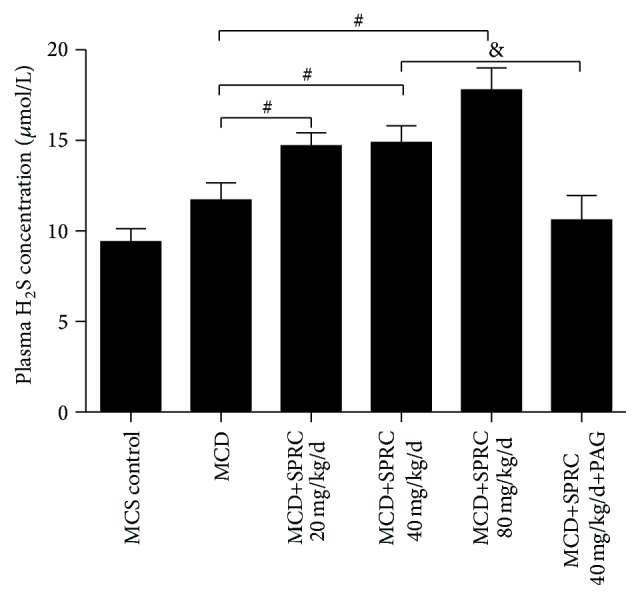
The effect of SPRC on endogenous H_2_S concentration. Mice fed a MCD diet were treated with SPRC (20, 40, and 80 mg/kg/d) and SPRC+PAG for four weeks. Data shown are means ± SEM; ^#^ *P* < 0.05 compared with MCD diet control group, *n* = 8; ^&^ *P* < 0.05 compared with the MCD+SPRC 40 mg/kg/d+PAG group, *n* = 8.

The results in Figure 4 showed that SPRC significantly elevated plasma concentration of H_2_S in MCD+SPRC (20, 40, and 80 mg/kg/d) groups except the PAG+SPRC-treated group.

### 3.5. SPRC Enhanced Cell Viability and Lowered Intracellular Lipid and ROS Accumulation Induced by OA in HepG2 Cells

**Figure 5:**
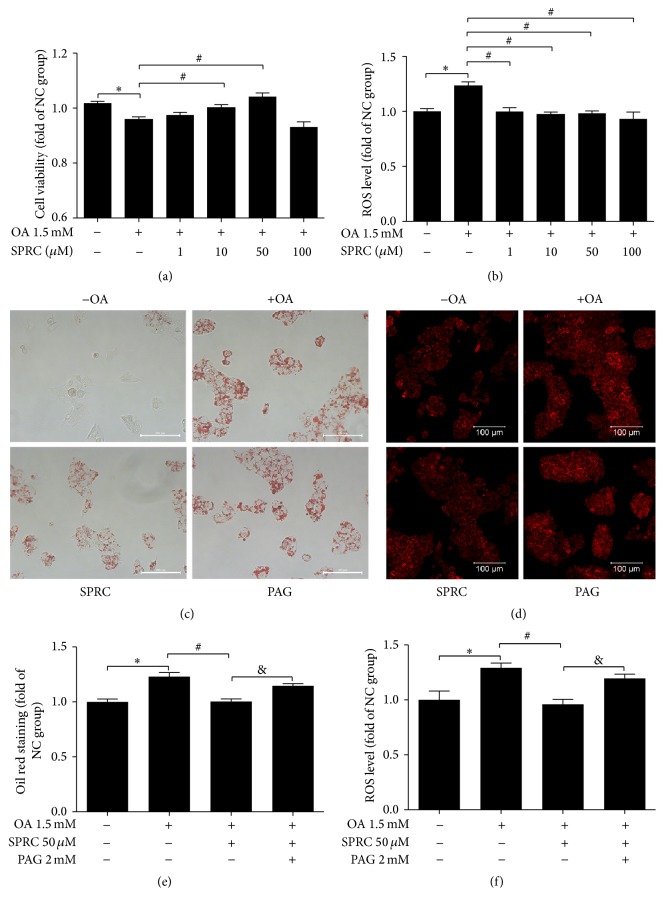
The effects of SPRC on cell viability, lipid contents, and ROS level in HepG2 cells. (a) HepG2 cells were incubated with different SPRC concentrations (1, 10, 50, and 100 *μ*M) for 6 h and then stimulated with OA (1.5 mM) for 18 h. Cell viability was measured by CCK-8 assay at the end of the treatment. (b) The same treatment was done as the viability detection, and ROS level was measured by microplate reader. The data represents mean ± SD. ^*∗*^ *P* < 0.05 compared with nontreated control; ^#^ *P* < 0.05 compared with OA-treated control. (c) Cells were incubated with or without PAG (2 mM) for 30 min followed by SPRC (50 *μ*M) for 6 h. Thereafter, cells were then treated with OA (1.5 mM) for 18 h. After the treatment duration, cells were stained with oil red O dye. The representative images of cells were captured by microscope at ×200 magnification. (d) Cells were incubated with or without PAG (2 mM) for 30 min followed by SPRC (50 *μ*M) for 6 h. Cells were then treated with OA (1.5 mM) for 18 h. Representative images of ROS level via DHE fluorescence in HepG2 cells detected by a laser confocal microscope at the end of the treatment. (e) Oil red O stained cells were measured at 510 nm wave length by using microplate reader. Data represent mean ± SEM of three independent experiments. (f) Quantitative analysis for ROS level in HepG2 cells. The data represents mean ± SD. ^*∗*^ *P* < 0.05 compared with nontreated control; ^#^ *P* < 0.05 compared with OA-treated control; ^&^ *P* < 0.05 compared with OA plus PAG.

The HepG2 cell lines were applied to study the detailed molecular mechanism of SPRC on NAFLD. Oleic acid (OA, 1.5 mmol/L) was used as an inducer to cause cellular oxidative stress in HepG2 cells. As shown in Figure 5(a), OA treatment significantly decreased the cell viability and increased intracellular ROS level. Meanwhile, different SPRC concentrations (1, 10, 50, and 100 *μ*mol/L) were preadded to the media of SPRC group for 6 h, followed by OA treatment for 18 h. The results in Figure 5(a) showed that SPRC (10, 50 *μ*mol/L) remarkably enhanced cell viability, compared to the OA-treated group. In addition, intracellular total ROS level was also significantly decreased by SPRC (10, 50, and 100 *μ*mol/L) (Figure 5(b)). To assess the effect of SPRC on lipid accumulation, we used oil red O staining to measure lipid content and obtained visual images. Cells were preincubated with or without PAG (2 mmol/L) for 30 min followed by SPRC (50 *μ*mol/L) for 6 h. Cells were then treated with OA (1.5 mmol/L) for 18 h. As shown in Figure 5(e), SPRC (50 *μ*mol/L) markedly aggravated the hepatic lipid storage induced by OA, while this effect was reversed by PAG (2 mmol/L) pretreatment. OD values were measured at 510 nm by the microplate reader. The representative images were obtained and shown in Figure 5(c). As shown in Figure 5(f), OA increased the ROS level, while SPRC reduced the ROS level in the OA-induced HepG2 cells. PAG (2 mmol/L, CSE inhibitor) pretreatment for 30 min inhibited the effect of SPRC (50 *μ*mol/L) on ROS clear. The representative images were shown in Figure 5(d).

### 3.6. SPRC Decreased Intracellular TG and MDA Accumulation and Enhanced SOD Activity in HepG2 Cells

**Figure 6:**
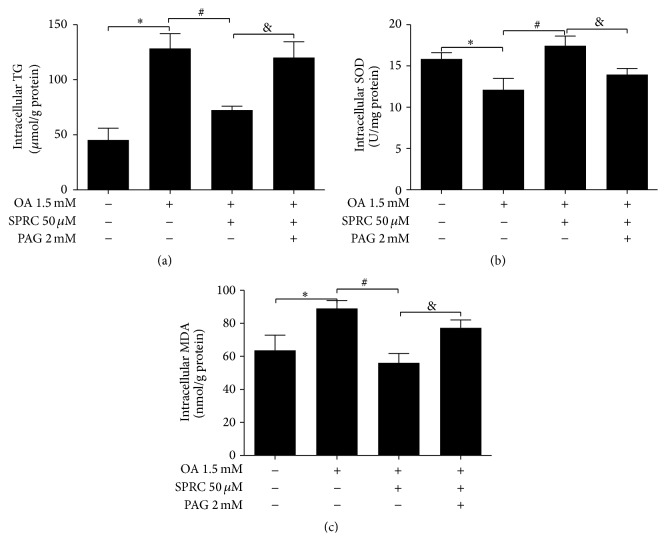
Effects of SPRC on TG, MDA accumulation, and SOD activity. (a) Cells were incubated with or without PAG (2 mM) for 30 min followed by SPRC (50 *μ*M) for 6 h. Cells were then treated with OA (1.5 mM) for 18 h. After the treatment duration, intracellular TG content was measured by TG testing kit. (b) Cells were treated as described before, and then SOD activity was detected by kit. (c) Cells were incubated in the same way as the MDA testing, and intracellular MDA was measured by MDA testing kit. The data represents mean ± SD. ^*∗*^ *P* < 0.05 compared with nontreated control; ^#^ *P* < 0.05 compared with OA-treated control; ^&^ *P* < 0.05 compared with OA plus PAG.

To determine the effect of SPRC on TG and MDA accumulation, as well as SOD activity, TG, MDA, and SOD testing kit were used to measure the lipid content and activity changes of SOD. Cells were incubated with or without PAG (2 mmol/L) for 30 min, followed by SPRC (50 *μ*mol/L) for 6 h. Cells were then treated with OA (1.5 mmol/L) for 18 h. As shown in Figures 6(a) and 6(b), SPRC (50 *μ*mol/L) significantly decreased the hepatic TG level and enhanced SOD activity in HepG2 cells induced by 1.5 mmol/L OA, while the effects were reversed by PAG (2 mmol/L) pretreatment. In addition, MDA accumulation was also significantly reduced in the SPRC group compared to the control group. And this effect was also reversed by the PAG pretreatment (Figure 6(c)).

### 3.7. SPRC Regulated ROS-Related mRNA Levels

**Figure 7:**
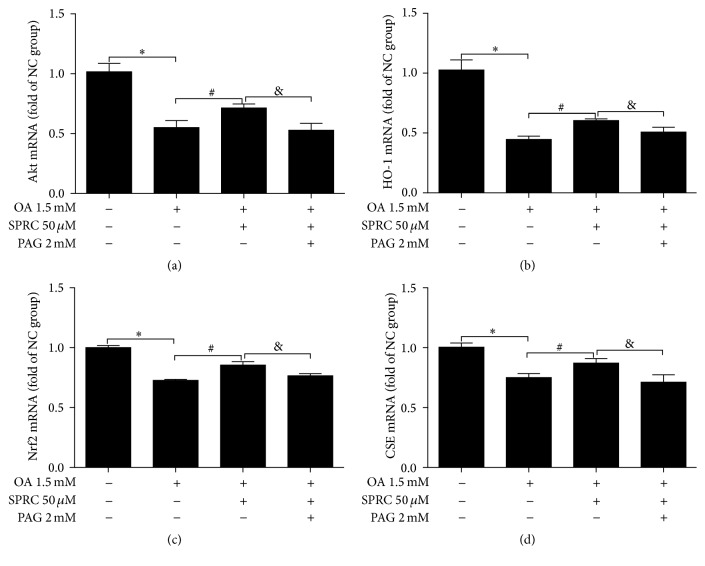
Regulation of SPRC on ROS-related genes. (a) Real-time PCR assays for Akt, HO-1, and Nrf2. (b) Real-time PCR assays for HO-1. (c) Real-time PCR assays for Nrf2. (d) Real-time PCR assays for CSE. The mRNA level of GAPDH was used as a reference for data normalization. The data represents mean ± SD. ^*∗*^ *P* < 0.05 compared with nontreated control; ^#^ *P* < 0.05 compared with OA-treated control; ^&^ *P* < 0.05 compared with OA plus PAG.

Because the role of SPRC in ROS is clear, we examined the effects of SPRC on the expression of these vital genes in HepG2 cells induced by OA. Cells were incubated with or without PAG (2 mmol/L) for 30 min followed by SPRC (50 *μ*mol/L) for 6 h. Cells were then treated with OA (1.5 mmol/L) for 18 h. The decreases in mRNA levels of Akt, HO-1, and Nrf2 were observed in the OA-induced HepG2 cells. However, treatment with SPRC increased mRNA levels of these three genes, as assessed by quantitative real-time PCR assays (Figures 7(a), 7(b), and 7(c)). In addition, SPRC upregulated the CSE mRNA level, which was reduced by OA treatment (Figure 7(d)). Finally, PAG treatment reversed all these effects of SPRC, as shown in Figure 7.

### 3.8. SPRC Activated PI3K/Akt Signaling Pathway in HepG2 Cells

**Figure 8:**
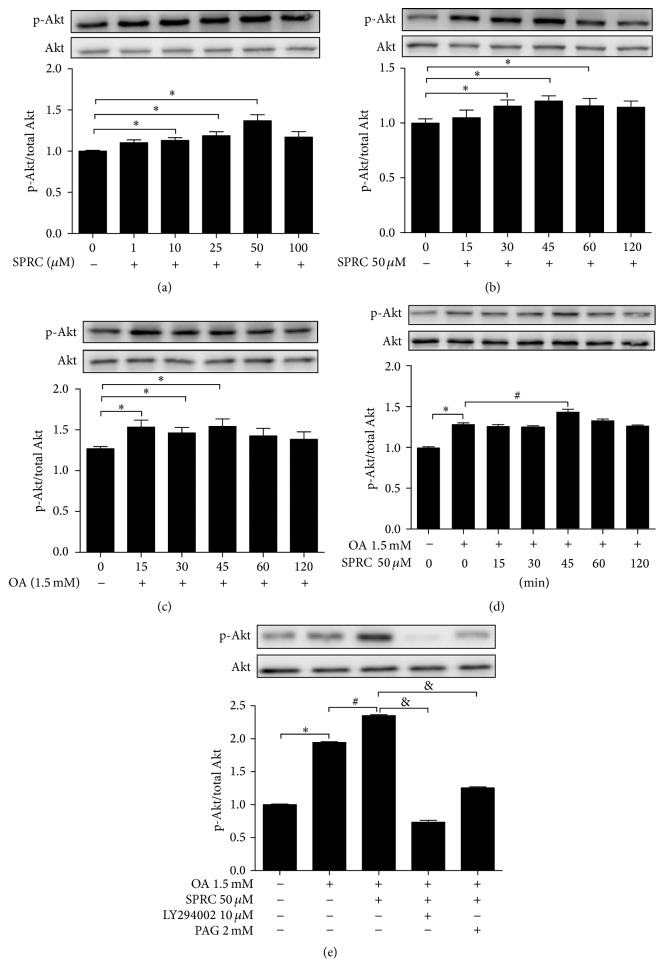
SPRC-induced Akt phosphorylation in OA-stimulated HepG2 cells. (a) Cells were incubated with indicated concentrations of SPRC (0, 1, 10, 25, 50, and 100 *μ*M) for 30 min. Western blot analysis gave the related ratio of p-Akt/Akt. (b) Cells were treated with SPRC (50 *μ*M) for indicated time periods. The densitometric analysis gave the related ratio of p-Akt/Akt. (c) Cells were only stimulated with OA (1.5 mM) for indicated time periods. Western blot analysis gave the related ratio of p-Akt/Akt. (d) Cells were preincubated with or without 50 *μ*M SPRC for a period of indicated times before addition of 1.5 mM OA for another 15 min. The densitometric analysis gave the related ratio of p-Akt/Akt. (e) Cells were preincubated with 10 *μ*M LY294002 or 2 mM PAG for 30 min before adding SPRC (50 *μ*M) for 30 min and. subsequently, stimulated with OA for another 15 min. The densitometric analysis gave the related ratio of p-Akt/Akt. Data represent mean ± SEM of three independent experiments. ^*∗*^ *P* < 0.05 compared with nontreated control; ^#^ *P* < 0.05 compared with OA-treated control; ^&^ *P* < 0.05 compared with OA plus PAG or LY294002.

To investigate whether SPRC can activate PI3K/Akt pathway and affect OA-induced Akt phosphorylation, HepG2 cells were incubated with indicated concentration of SPRC (0, 1, 10, 25, 50, and 100 *μ*mol/L) for 30 min. The results in Figure 8(a) showed that SPRC significantly increased phosphorylation of Akt in a concentration-dependent manner, with peak level of p-Akt observed at 50 *μ*mol/L incubation, followed by a drop at 100 *μ*mol/L, compared with the nontreated group (Figure 8(a)). Incubation of HepG2 cells with 50 *μ*mol/L SPRC for indicated time periods induced phosphorylation of Akt in a time-dependent manner during the time periods shown in Figure 8(b). In particular, the Akt phosphorylation reached the peak value at 45 min and then decreased slightly at 60 and 120 min (Figure 8(b)). In addition, as shown in Figure 8(c), incubation with only OA (1.5 mmol/L) also increased the p-Akt at 15, 30, and 45 min, with a peak value at 15 min. Furthermore, cells were also pretreated with SPRC (50 *μ*mol/L) for indicated time periods (0, 15, 30, 45, 60, and 120 min) and then stimulated by OA for another 15 min. The results in Figure 8(d) showed that the phosphorylation of Akt was significantly induced by SPRC pretreatment compared to the OA-treated control cells at 45 min. Importantly, SPRC-induced Akt phosphorylation was blocked by the PI3K inhibitor LY294002 (10 *μ*mol/L) or CSE inhibitor PAG (2 mmol/L) (Figure 8(e)). The total protein levels of the Akt were not affected by SPRC treatment. Thus, these data indicated that SPRC caused Akt phosphorylation involvement of activation of the PI3K pathway.

### 3.9. SPRC Regulated HO-1 Expression via Activation of Akt/Nrf2 Pathway in HepG2 Cells

**Figure 9:**
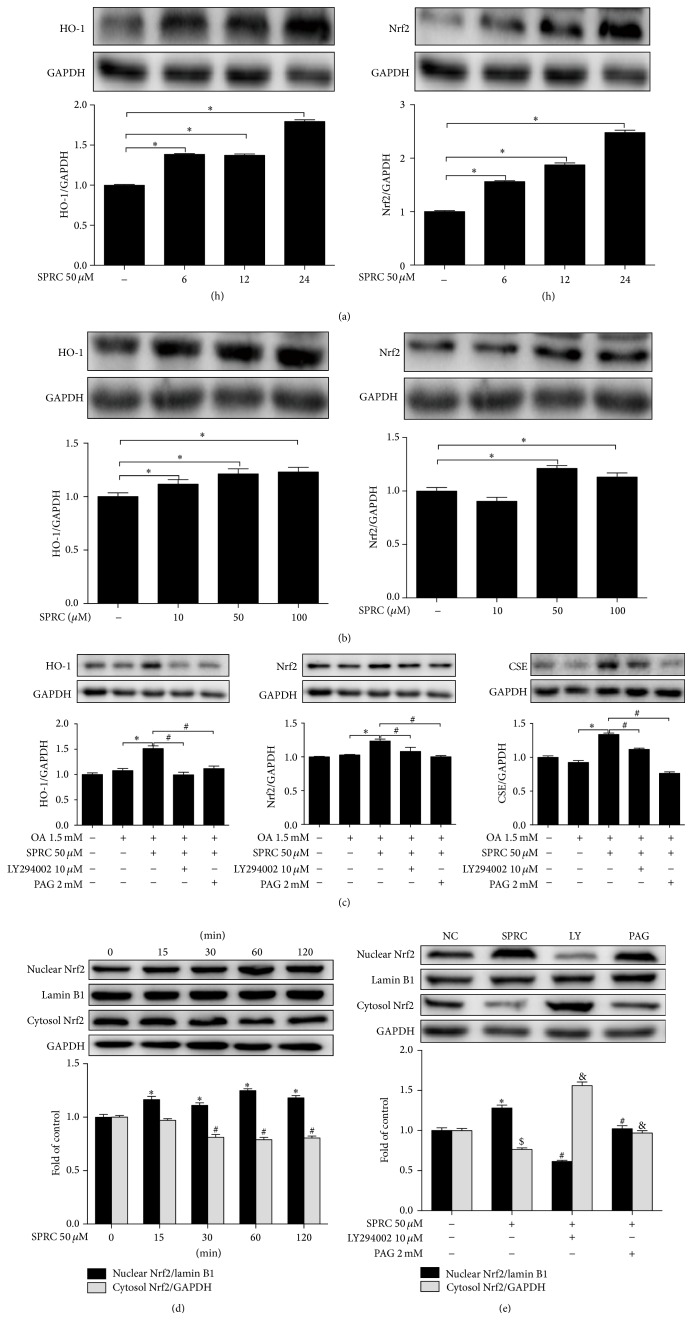
SPRC upregulated HO-1 expression via activation of Akt/Nrf2 pathway in HepG2 cells. The cells were incubated with SPRC (50 *μ*M) for indicated periods (a) or indicated concentrations of SPRC for 24 h (b), and the expression of HO-1 and Nrf2 was detected by Western blot, and GAPDH was used as a loading control; data shown are means ± SEM, ^*∗*^ *P* < 0.05 compared with untreated cells; data were from at least three independent experiments, each performed in duplicate. (c) The cells were pretreated with LY294002 or PAG for 30 min, then incubated with SPRC (50 *μ*M) for 6 h, and then stimulated with or without OA (1.5 mM) for 18 h, and the expression of HO-1 and Nrf2 was detected by Western blot, and GAPDH was used as loading control; data shown are means ± SEM; ^*∗*^ *P* < 0.05 compared with untreated cells; ^#^ *P* < 0.05 compared with OA-stimulated cells; data were from at least three independent experiments, each performed in duplicate. (d) The cells were treated with SPRC (50 *μ*M) for indicated periods (0, 15, 30, 60, and 120 min), and the translocation of Nrf2 from cytosol to nucleus was detected by Western blot, and Lamin B1 was used as loading nuclear control and GAPDH was used as loading cytosol control; data shown are means ± SEM; ^*∗*^ *P* < 0.05 compared with nuclear Nrf2 in untreated cells; ^#^ *P* < 0.05 compared with cytosol Nrf2 in untreated cells; data were from at least three independent experiments, each performed in duplicate. (e) The cells were pretreated with LY294002 and PAG for 30 min and then incubated with SPRC (50 *μ*M) for 60 min, and the translocation of Nrf2 from cytosol to nucleus was detected by Western blot, and Lamin B1 was used as loading nuclear control and GAPDH was used as loading cytosol control; data shown are means ± SEM; ^*∗*^ *P* < 0.05 compared with nuclear Nrf2 in untreated cells; ^#^ *P* < 0.05 compared with nuclear Nrf2 in SPRC-treated cells; ^$^ *P* < 0.05 compared with cytosol Nrf2 in untreated cells; ^&^ *P* < 0.05 compared with cytosol Nrf2 in SPRC-treated cells; data were from at least three independent experiments, each performed in duplicate.

Upregulation of HO-1 expression played a vital role in protecting cells against the cellular injury induced by oxidative insults. To elucidate the effect of SPRC on the expression of HO-1 in HepG2 cells, the cells were incubated with SPRC (50 *μ*mol/L) for various periods (6, 12, and 24 h) or indicated concentrations (10, 50, and 100 *μ*mol/L) for 24 h. In addition, the expression of HO-1 and Nrf2 was assessed by Western blot. As shown in Figure 9(a), the expressions of HO-1 and Nrf2 in the cells treated with SPRC (50 *μ*mol/L) significantly increased at 6 h treatment compared to the nontreated cells with a peak value at 24 h. Data were obtained from at least three independent experiments, each of which was performed in duplicate. Cells were also incubated with different concentrations of SPRC for 24 h, resulting in increases in HO-1 and Nrf2 protein in a concentration-dependent manner. The peak values of HO-1 and Nrf2 protein were both observed at the concentration of SPRC 50 *μ*mol/L. In addition, HO-1 increased at all SPRC concentrations (10, 50, and 100 *μ*mol/L) as shown in Figure 9(b). However, Nrf2 was enhanced only at SPRC concentrations of 50 and 100 *μ*mol/L (Figure 9(b)). To examine the possible signaling pathways involved in HO-1 induction, the cells were pretreated with a PI3K inhibitor LY294002 (10 *μ*mol/L) or PAG (2 mmol/L) for 30 min and then incubated with SPRC (50 *μ*mol/L) for 6 h, followed by OA stimulation for another 18 h. As shown in Figure 9(c), the expressions of HO-1, Nrf2, and CSE in SPRC-treated cells were notably induced compared to the OA-treated control cells, while LY 294002 and PAG blocked this effect of SPRC. In addition, activation of PI3K/Akt signaling also led to translocation of factor Nrf2, which further resulted in the upregulation of HO-1 expression. As shown in Figure 9(d), Nrf2 translocation in the cells treated with SPRC (50 *μ*mol/L) was significantly induced from cytosol to nucleus in a time-dependent manner. The Nrf2 translocation reached the peak value at 60 min and dropped slightly at 120 min. However, the effect of SPRC was abolished in the presence of LY294002 or PAG (Figure 9(e)). Therefore, the results indicated that the upregulation of HO-1 by SPRC was dependent on PI3K/Akt pathway activation and Nrf2 nuclear translocation, which resulted in HO-1 induction.

### 3.10. Induction of HO-1 by SPRC Was Critical for Its Antioxidative Activity in HepG2 Cells

**Figure 10:**
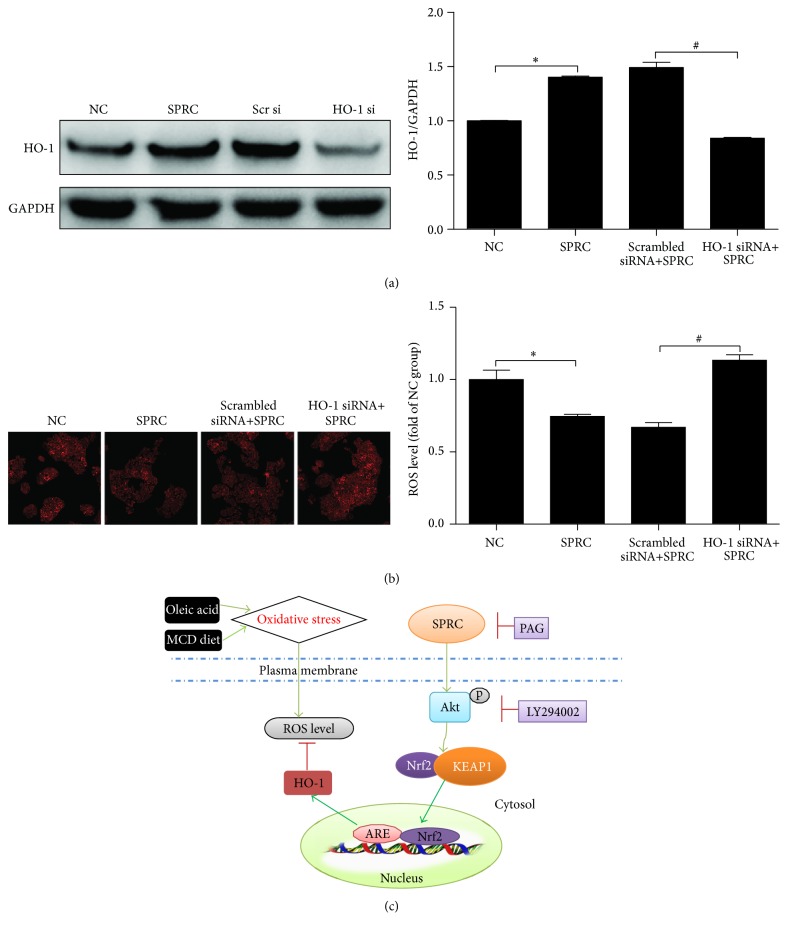
Induction of HO-1 by SPRC was critical for its antioxidative activity in HepG2 cells. (a) The cells were incubated with HO-1 siRNA or scrambled siRNA and then incubated with SPRC (50 *μ*M) for 24 h, and the expression of HO-1 was detected by Western blot, and GAPDH was used as loading control; data shown are means ± SEM; ^*∗*^ *P* < 0.05 compared with nontreated control; ^#^ *P* < 0.05 compared with HO-1 siRNA. Data were from at least three independent experiments, each performed in duplicate. (b) The cells were incubated with HO-1 siRNA or scrambled siRNA and then incubated with SPRC (50 *μ*M) for 24 h. Representative images of ROS level via DHE fluorescence in HepG2 cells detected by a laser confocal microscope at the end of the treatment (original magnification, ×200). (c) Proposed cell signaling pathway for SPRC-induced p-Akt/Nrf2/HO-1-mediated cytoprotective effect. Green arrow presents stimulation and red bar indicates inhibition.

As shown in Figure 10(a), we confirmed our observations by using an HO-1 knockdown strategy. Transfection with HO-1 siRNA, but not scramble siRNA, abolished SPRC-induced HO-1 expression. Furthermore, the effect of SPRC on reducing intracellular ROS level disappeared in the HepG2 cells transfected with HO-1 siRNA (Figure 10(b)). Thus, the result indicated that HO-1 induction by SPRC was a critical step to provide antioxidative activity.

## 4. Discussion

NAFLD is characterized by oxidative stress (OS) and impaired utilization of lipid in tissue, leading to an increase in circulating lipid [1]. The present study was designed to determine the effects of SPRC on NAFLD using both an* in vivo* model and an* in vitro* model.

Satapati et al. and Surapaneni et al. reported that ROS, SOD, and MDA levels were changed during nonalcoholic fatty liver disease (NAFLD) [26, 27]. By using chemical kit, we found that SPRC could significantly regulate SOD activity and ROS and MDA levels in MCD diet-induced NAFLD mice. It should be noted that SPRC significantly reduced the hepatic MDA level at the dosages of 20 and 40 mg/kg/d. However, the hepatic MDA levels of MCD and MCD+SPRC 80 mg/kg/d groups seem to be equal. This result indicated that high dosage of SPRC is ineffective to MDA level. It is possible that the indicator (MDA) of lipid peroxidation is more sensitive to SPRC. Note that we found that only the first-week treatment of PAG abolished the effect of SPRC on weight regain in SPRC+PAG group. This discrepancy might be due to the complexity of the organism. It might be a challenging study to extensively illustrate how SPRC affects the weight. Previous studies indicated that HO-1 is one of the key enzymes related with OS in the liver [28] and plays a critical role in regulating hepatic ROS. The induction of HO-1 is principally regulated by the Nrf2/Kelch-like ECH-associated protein 1 (KEAP1) system, which is a main sensor system for antioxidative response and regulates expression of a series of antioxidant enzymes [29, 30]. In case of oxidative stress, cytosolic Nrf2 will depart from the Nrf2/KEAP1 complex, which then translocates to the nucleus. This leads to the binding of nuclear Nrf2 and antioxidant response element (ARE), resulting in triggering HO-1 expression [7, 29, 30]. It has been reported that Nrf2 is an important downstream target of PI3K/Akt activation that can lead to Nrf2 translocation from cytosol into nucleus and thus activate the ARE/HO-1 pathway [31, 32]. Taken together, we hypothesized that the effect of SPRC on NAFLD may be through Akt phosphorylation, Nrf2 translocation, and HO-1 expression. By Western blot, we detected the regulatory effect of SPRC on the expression of hepatic OS-related proteins (Akt, Nrf2, and HO-1). It should be noticed that total Akt was remarkably decreased in MCD group compared to the MCS group. This finding suggests that Akt is critical for the development of fatty liver.

The effect of SPRC on NAFLD and its underlying mechanism require further investigation. OA causes oxidative stress in hepatocytes, which can mimic the* in vivo* NAFLD situation in HepG2 cells [33]. The* in vitro* experimental results indicated that SOD activity and ROS and MDA levels were regulated by SPRC in OA-induced HepG2 cells, which matched the* in vivo* experimental results. To further explore the underlying mechanism of SPRC in OA-induced oxidative stress, Akt phosphorylation, Nrf2 translocation, and the expression of HO-1 were analyzed by Western blot. We found that the expression of these three proteins was significantly upregulated in OA-induced HepG2 cells. Interestingly, a PI3K inhibitor LY294002 significantly reduced the SPRC-induced translocation of Nrf2 to the nucleus and diminished the upregulation of HO-1. This result matches the preliminary report that Akt and Nrf2 play an important role in antioxidative effect of SPRC in high glucose-induced H9C2 cells [16]. Notably, the effect of SPRC on ROS elimination was abolished in the group transfected with HO-1 siRNA. Based on these findings, it can be deduced that the antioxidative activity of SPRC was mediated by the induction of HO-1, which was involved in the activation of PI3K/Akt pathway and translocation of Nrf2 from cytosol to nucleus.

SPRC has been reported to be a substrate of CSE that is the most significant enzyme for the generation of endogenous H_2_S in the liver [34, 35]. In addition, a number of studies have reported that H_2_S activates Akt/Nrf2/HO-1-dependent signaling [17, 36, 37]. In this study, we validated that PAG could reverse the effect of SPRC on ROS elimination, Akt activation, HO-1 induction, and Nrf2 translocation in the livers of MCD diet-induced mice and OA-induced HepG2 cells. Taking all these findings together, we could build a relationship between SPRC/CSE/H_2_S and PI3K/Akt/Nrf2/HO-1 pathways, which thus provide a new insight into the antioxidative defense mechanism.

Overall, our results clearly showed that SPRC not only alleviated MCD diet-induced NAFLD, but also attenuated OA-induced ROS level in HepG2 cells, which indicated that SPRC could be useful for the treatment of NAFLD.

## 5. Conclusions

In summary, we demonstrated that the novel chemical SPRC exerted antioxidative property on MCD diet-induced NAFLD through upregulation of HO-1, which was involved in PI3K/Akt pathway and Nrf2 translocation. Therefore, the results suggest that SPRC has the potential for beneficial therapeutic interventions for NAFLD.
